# Ethical aspects of sudden cardiac arrest research using observational data: a narrative review

**DOI:** 10.1186/s13054-018-2153-3

**Published:** 2018-09-13

**Authors:** Marieke A. R. Bak, Marieke T. Blom, Hanno L. Tan, Dick L. Willems

**Affiliations:** 1 Section of Medical Ethics, Department of General Practice, Amsterdam UMC, University of Amsterdam, Meibergdreef 9, 1105 AZ Amsterdam, The Netherlands; 2 Department of Cardiology, Heart Center, Amsterdam UMC, University of Amsterdam, Amsterdam, The Netherlands

**Keywords:** Ethics, Informed consent, Sudden cardiac arrest, Emergency research, Big data

## Abstract

Sudden cardiac arrest (SCA) accounts for half of all cardiac deaths in Europe. In recent years, large-scale SCA registries have been set up to enable observational studies into risk factors and the effect of treatment approaches. The increasing scale and variety of data sources, coupled with the implementation of a new European data protection legal framework, causes researchers to struggle with how to handle these ‘big data’. Data protection in the SCA setting is especially complex since patients become at least temporarily incapacitated, and are thus unable to provide prospective informed consent, and because the majority of patients do not survive. A narrative review employing a systematic literature search was conducted to thematically analyse ethical aspects of non-interventional emergency medicine and critical care research. Although the identified issues may apply to a wider patient population, we describe them within the context of SCA research. Potential harms were found to include: privacy breaches, genetic discrimination and issues associated with the disclosure of individual findings, study design and application of research results. Measures proposed to mitigate harms were: alternative informed consent models including deferred or waived consent and data governance approaches promoting data security, responsible sharing and public engagement. The themes identified in this study may serve as a basis for a much-needed ethical framework regarding research with data from patients with acute and critical illness such as SCA.

**Electronic supplementary material:**

The online version of this article (10.1186/s13054-018-2153-3) contains supplementary material, which is available to authorized users.

## Background

Sudden cardiac arrest (SCA) is lethal within minutes if left untreated. Despite advances in emergency medicine, SCA remains a major public health concern with global average survival rates reported to be only 7% [1]. The same study found that in Europe, SCA accounts for approximately 50% of cardiac deaths and 20% of all natural deaths. Designing effective individualised prevention and innovative treatment strategies requires insight into causative factors of SCA and the effect of (first-response) treatment approaches. Since the causes of SCA are complex, and patients have unique sets of causative factors, this necessitates large numbers of study participants. Thus, there is a long-recognised need for epidemiological database research in order to improve SCA prevention and care [2, 3].

Non-interventional, observational research can produce evidence matching the strength of interventional studies while providing several advantages in terms of external validity, cost and ethics [4, 5]. By informing the development of personalised prevention and treatment approaches, observational studies may lead to better outcomes for patients as well as cost savings in healthcare [6].

In recent years, various large observational SCA cohorts have been established around the world and international research collaborations have emerged [7–10]. In addition to collecting data from Emergency Medical Services and hospitals, some registries contain additional data from general practitioners and pharmacists as well as socio-economic data initially gathered for administrative purposes (see for instance the Dutch ARREST study [11]). It is also increasingly common in critical illness research to establish biobanks with DNA specimens. These might be obtained from blood samples either collected specifically for research purposes or derived from residual blood in samples routinely collected for patient care. In some studies, DNA from patients who die on the scene is extracted from endotracheal intubation tubes collected from ambulance posts or emergency rooms [11]. Due to the transient and transdisciplinary nature of emergency medical care, SCA research data generally come from multiple data sources and are subsequently ‘linked’ [12].

This increasing scale and variety of data, accelerated by the emergence of more affordable techniques, e.g. in the field of DNA sequencing, causes data to be increasingly ‘big’ in SCA research specifically and cardiovascular research in general [13, 14]. However, the collection and use of big observational data in healthcare brings about a number of ethical dilemmas about the appropriate balance between medical research and data subjects’ rights [15].

In SCA research, these issues are intensified by the emergency circumstances that prevent patients from giving prior informed consent to the use of their data, which is especially problematic for the majority of patients who do not survive. Although ethical issues in SCA clinical trials have been widely debated [16, 17], there is a paucity of literature on the ethical aspects of non-interventional research with data obtained from patients in emergency circumstances [18]. Moreover, the debate tends to be focused on informed consent, while there might be other issues that researchers should take into account when conducting database studies, especially in this age of globalisation, genomics and big data. Guidance on these issues would be particularly timely given the implementation of a new European Union (EU) data protection regulatory framework on 25 May 2018.

In the European General Data Protection Regulation 2016/679 (GDPR), personal data are defined in Article 4(1) as “any information relating to an identified or identifiable natural person (‘data subject’)”. The GDPR considers genetic, biometric and health data to be sensitive data for which strict rules apply (Table 3 presents an overview of the key changes in the EU data protection law that are relevant for research with medical data). Some researchers have worried that medical research may be hampered by ‘data overprotection’, fearing that new EU data protection law would require written consent for all research uses [19]. In response to criticism, the text of the GDPR was amended to reflect the importance of processing of sensitive data for research purposes. In the final version of the law, informed consent is only one of several grounds that can be used to justify the lawfulness of processing sensitive data, while the need for data for scientific purposes is another (Article 9(2)) [20]. Concerns about a potentially detrimental impact of data protection legislation on observational research have also been voiced in other countries, for instance in the United States where the Privacy Rule (as an addendum to the Health Insurance Portability and Accountability Act) governs the use of identifiable health information for research [21], or in Canada with the Personal Information Protection and Electronic Documents Act [22].

To date, no comprehensive review has been conducted on the ethical aspects germane to observational, non-interventional research into SCA or other acute and critical conditions. The present study was conducted as part of a newly established European consortium called ESCAPE-NET (European Sudden Cardiac Arrest network towards Prevention, Education and New Effective Treatment) [10]. The aim of this paper is to support the ethical conduct of non-interventional SCA database research, such as ESCAPE-NET, by reviewing key ethical aspects from the literature. It should be noted that the reviewed literature is used to highlight key issues in research with data from SCA patients in particular, although our study findings will also apply to other groups of acutely and critically ill patients.

### Literature search

We performed a systematic search with terms and MeSH headings related to ‘sudden cardiac arrest’ or ‘emergency medicine’, ‘ethics’ and ‘observational research’. Five databases were searched: PubMed, CINAHL, Philosopher’s Index, Philpapers, and Web of Science. Reference lists of retrieved articles were hand-searched to identify additional publications. The search was performed in December 2017 and was initially limited to English language articles that mentioned ethical aspects of observational studies in SCA specifically. However, the focus of the literature search was extended from SCA to emergency medicine and critical care in general, after the initial sample was found to be rather small. Studies were excluded that discussed ethical aspects of data processing in an interventional research setting or care context. For the search strategy, see Additional file 1: Table S1. Due to the limited number and great heterogeneity of articles retrieved, we chose not to present the results as a systematic review. Instead we extracted ethical themes from the retrieved articles and used those as a basis for a narrative review. The thematic narrative is supplemented by literature on the ethics of research with (bio-) medical data in general to provide context and further understanding of each ethical theme.

## ‘First, do no harm’: potential risks to patients

Ethical themes from the retrieved articles are sorted into two main clusters describing (1) potential harms to patients and (2) measures that researchers can take to mitigate these harms (Table 1). In the following sections we discuss potential harms of SCA data research.

**Table 1:** Clustering of ethical aspects related to SCA research with observational data

Cluster	Ethical theme	Examples from the literature	References
Potential harms	Privacy	• Linkage across datasets in critical care research increases the risk of re-identification of the individual	[41]
	Genetic discrimination	• Genetic testing for SCA-associated conditions creates the potential for stigma and discrimination	[30, 31]
	Disclosure of individual findings	• Dilemma: whether or not to inform patients with a high risk of SCA who refuse to know their test results	[36]
	Research design	• SCA data may be of low quality due to the acute setting and variety of data sources: sound methodology is vital	[38–42]
		• Incorrect subject selection may exacerbate SCA knowledge gaps between developed and developing countries	
	Applications	• The creation of (incorrect) risk profiles (e.g. for hypertrophic cardiomyopathy) may give rise to health disparities	[41, 46]
Protective measures	Informed consent	• Insistence on informed consent for use of data from emergency medical settings would bias research	[18, 41, 53–55, 60]
		• Deferred consent for data collection is seen by patients as an acceptable consent model in emergency settings	
	Data governance	• Critical care research without consent requires safeguards (e.g. safe-havens) to protect data security	[41, 54]

### Privacy of personal data

Researchers should ensure adequate protection of participants’ personally identifiable data. This right to data protection as described in the GDPR is based in part on the right to privacy [23], which is enshrined in Article 8 of the European Convention of Human Rights. The intrinsic value of privacy arises from the individual’s need to freely relate to others in a social context and to have the ability to control who gets access to one’s personal information [24]. Additionally, privacy is valuable since it promotes other fundamental values such as personal autonomy, individuality, human dignity and respect for persons [25].

Privacy rights can be violated even without the subject’s knowledge. For instance, participants’ personal information may be used for *secondary* research that goes against their religious beliefs. If knowledge of the harm is not a necessary condition for harm, then deceased SCA victims can be harmed in terms of their ‘ante-mortem’ person’s rights or their legacy [26]. However, the discussion on post-mortem privacy is still unresolved, and few guidelines address the rights of deceased persons. The European Court of Human Rights only recognises privacy rights for the deceased when they are connected to living individuals, as is the case with genetic data which may contain sensitive information about living relatives [26].

A review commissioned by the Nuffield Council on Bioethics showed that evidence of direct harms to wellbeing resulting from data breaches in healthcare organisations is rare, possibly due to anecdotal reporting [27]. At a minimum, however, data breaches would cause distress among patients and impact on participants’ trust in medical institutions, negatively affecting support for research and people’s willingness to participate [28]. What is more, confidentiality breaches involving genetic research data may lead to worries of discrimination if third parties (e.g. insurance companies) gain access to them, as discussed in the following section.

### Genetic discrimination

Even though discrimination based on genetic features is prohibited in the EU Charter of Fundamental Rights and the Council of Europe’s Oviedo Convention on Human Rights and Biomedicine, genetic discrimination by family members, employers and insurers is widespread [29]. Fears of discrimination were documented in Dutch families involved in genetic testing for hypertrophic cardiomyopathy, a common cause of SCA in young people [30]. In North America, Mohammed et al. [31] showed that increased genetic testing negatively impacted insurability for patients with sudden arrhythmic death syndromes. Discrimination does not only harm people’s dignity but also leads to direct harms to health [29]. For instance, limited employability or insurability might create financial hardship which potentially increases SCA risk further, since lower socio-economic status is associated with a higher incidence of SCA [32]. Moreover, worries about the discriminatory use of genetic data have been shown to deter people from participation in medical research, thus possibly impeding SCA research progress [33].

### The (non-) disclosure of individual findings

Physicians have a professional and moral duty to warn their patients about potential threats to health. Recent studies and guidelines have expanded this duty to researchers in the context of disclosing actionable genetic findings to study participants [34, 35]. However, research participants may not want to know about any genetic findings. Pullman and Hodgkinson [36] describe a case of genetic research into autosomal dominant arrhythmogenic right ventricular cardiomyopathy, a cause of SCA in young people that can be effectively treated. Several subjects with a DNA variant causing high risk of premature death had refused to know their results and died of preventable SCA. The authors state that it is “unethical for genetic researchers to absolve themselves of clinical responsibilities for research subjects and/or their families” and stress the importance of advance arrangements in genetic SCA studies, i.e. not enrolling subjects if they do not want to receive results. Although a full discussion of the ‘right not to know’ is beyond the scope of this paper, this case study helps to show that not using data may also have harmful consequences.

### Fairness in research design

For any type of study, it is unethical to use resources and risk subjects’ privacy when there is no sound methodology [37]. Factors such as bias and confounding should be taken into account especially because in SCA research, data may be of lower quality due to the emergency setting. SCA data are often not gathered for research purposes, and variation in registration or reporting of SCA may lead to errors and bias when combining databases [38–40]. These limitations might be amplified in studies with very large datasets [41].

At a global level, it is important that research is conducted not just in developed countries, which would exacerbate existing knowledge disparities with less-developed countries, and that the results benefit those groups of patients who have contributed their data. SCA populations from different ethnic backgrounds and geographic areas are epidemiologically distinct, and local emergency medicine research in low- and middle income countries is needed, as described by Aufderheide et al. [42].

### Responsible application of research results

Research into the causal basis of SCA may lead to the development of new genetic tests and risk stratification algorithms, and future cost reductions may stimulate health policy makers to implement population-wide screening for SCA risk [43, 44]. Rumsfeld et al. [13] note that justice issues may arise if screening data are used to differentially provide care to those at high risk of cardiovascular disease, or when people who opt out of data collection would lose the right to benefit from the results of research. Moreover, research on SCA risk factors could lead to health promotion practices that may be seen as a form of control over the individual [45].

In addition to the potential for individual discrimination, group-level harms may arise when risk profiles are created for certain (racial) groups, especially when done incorrectly. Manrai et al. [46] have reported genetic misclassification of common DNA variants among inhabitants of the USA with African ancestry as pathogenic variants predisposing them for hypertrophic cardiomyopathy, thereby potentially creating further health disparities between black and white Americans. The use of genetic data and the linkage across datasets may thus increase the potential for stigmatisation of ethnic minorities [47]. Although these are largely theoretical possibilities, it is important that researchers are aware of these potentially harmful effects and consequently adopt appropriate measures to protect subjects’ rights.

## Measures to protect SCA patients in observational studies

The following sections provide an overview of ethically acceptable approaches to research with patient data in the context of medical emergencies such as SCA. We first describe informed consent, and subsequently other data governance practices.

### Informed consent in the emergency medical setting

In light of the potentially harmful consequences of research with SCA data, participants ought to be granted some form of control over the uses of information about them, which may be realised by an informed consent process. Voluntary informed consent is grounded in the right to self-determination and has been stressed in the Nuremberg Code and the Declaration of Helsinki, as well as in the International Ethical Guidelines for Epidemiological Studies published by the Council for International Organisations of Medical Sciences [48].

Exceptions to prospective consent are important in SCA research because a majority of patients do not survive the SCA episode, while others become incapacitated as a result of it. The problem with obtaining SCA patients’ consent has been widely debated in the context of clinical studies in which interventions are performed [49, 50]. However, we found little discussion in the literature about the ethics of non-interventional emergency research. Nonetheless, it is clear that insistence on seeking consent for SCA data use would pose not only practical problems but also ethical ones, two in particular.

1. Requiring informed consent for studies using data from SCA patients would exclude the majority of patients who cannot consent and would thereby bias research. Such *consent bias* causes societal harm by hampering medical research and skewing the data [51].
2. Not using data from people unable to consent would violate some patients’ wishes. Denying participation to deceased SCA victims and to those who lack the mental capacity to consent deprives them of the opportunity to contribute to research and thereby benefit society [18].

The first point relates to the scientific validity of the research. Consent bias would render the study findings less reliable and thereby potentially harm future patients, while also devaluing the contribution of those who did consent [52]. Tu et al. [53] describe the problems with informed consent in the Canadian Stroke Registry where less than half of potential participants consented to the use of their data due to difficulties with obtaining consent (i.e. people died or became incapacitated but had no surrogate decision maker). The resulting registry was not representative of the typical stroke patient: participants in the database were younger and more likely to be alert at admission and alive at discharge. Therefore, in a consecutive phase the ethics committees of the participating centres approved data collection without consent [54]. Consent bias can also arise from different attitudes towards research among ethnic or cultural groups: Freeman et al. [55] found that African American surrogate decision makers of critically ill patients were less receptive of genetic data collection than their counterparts of European descent.

The second point concerns patients’ right to self-determination. For some people, sharing their data for research purposes is an important goal. Informed consent should thus not be interpreted only as a means to protect research subjects from harm, but also as an “act of autonomy that has a positive motive (e.g., compassion)”, as stated by Christen et al. [56]. Denying the possibility of contributing data for those unable to consent would be based on the *assumption* that the patient places individual privacy concerns above health benefits for others.

#### Alternatives to prospective consent: waived or deferred

Considering the above, researchers seeking to use SCA data need to find ethically acceptable alternatives to prospective consent. Designing informed consent processes can be a complex endeavour, considering the various types of consent models (Table 2). The largest problem within emergency medicine research is the timing of consent: since prior consent is impossible, one could either apply for an *exception* from consent or seek *deferred* consent.

**Table 2:** Informed consent models

	Model	Description	Advantages	Disadvantages
Level of control	Opt-in	Actively given, explicit consent	- Promotes autonomy - Respects patients’ expectations and preserves trust	- Lower response rates and potential consent bias - Relatively costly and time-consuming
	Opt-out	Consent is presumed unless participant objects	- Higher participation rates and less bias than opt-in - More practical, less costly	- Assumes that people want to participate: may infringe upon autonomy - Potentially less informed
	No consent	Study is conducted without consent (exception/waiver)	- Maximum participation rates and no consent bias - Most practical, least costly	- No control whatsoever by data subjects: least autonomy-enhancing
Timing	Prospective	Consent is given prior to the start of data collection	- Promotes autonomy - Respects patients’ or representatives’ expectations and preserves trust	- Time pressure and stress in emergencies: consent not fully informed/valid - Excluding (temporarily) incapacitated subjects causes bias and may not respect subjects’ wishes
	Deferred	Retrospective consent which is sought after data collection	- Provides temporarily incapacitated subjects the opportunity to participate - More valid than prior (subject or representative) consent in stressful situations	- Logistical issues with reaching participants - Data are already collected: less autonomy-enhancing than prior consent
Specificity	Study-specific	Consent for the use of data for one specific aim	- Promotes autonomy since patient has a high level of control over uses	- Requires re-contacting subjects for new aims: logistical challenge and burden for participants - Bias when contact attempts are unsuccessful
	Tiered	Subject chooses from a list what types of research are allowed (online: dynamic consent)	- Promotes autonomy since patient has a high level of control over uses	- Burdensome and complicated for subjects: requires detailed understanding
	Broad or blanket	Consent for overall research topic (broad) or without limitation (blanket)	- Smallest burden for researchers and patients in terms of re-contacting	- Broad consent may not be truly informed - Blanket consent often not accepted by research ethics committees

Clinical registries can be set up without the requirement for individual patient consent. Provided that appropriate safeguards are in place, an exception from consent is often seen as permissible because of the large benefits and small risks, coupled with the aforementioned issues associated with seeking consent. However, according to Tu et al. [53] certain components (e.g. data linkage and genetic information) of acute illness database research add new responsibilities for which consent should be obtained if possible. Namely, data linkage creates highly specific knowledge on individuals, thereby causing a higher risk of re-identification [57]. DNA sequences are uniquely identifiable due to the presence of individual genetic markers, which have been used to re-identify anonymous DNA donors in a study by Gymrek and colleagues [58], and they reveal information about not only the individual but also their relatives.

SCA survivors may be retrospectively asked permission for data collection, a practice known as deferred consent [59]. When the subject becomes mentally incapable of giving consent as a result of the SCA, the researchers must seek consent from the legally authorised representative (‘proxy’ or ‘surrogate’), according to Article 30 of the Declaration of Helsinki. Because proxy consent during the emergency is of questionable value due to the emotional nature of the situation, it is generally sought later as well [52]. In a study by Fox et al. [18] where residual blood was collected from trauma patients, deferred consent was seen by patients and legally authorised representatives as an acceptable approach, with no refusals to consent. Deferred consent may be obtained through telephone conversation, which was found by Offerman et al. [60] to be highly effective and well-received among trauma patients. The time until seeking consent should be long enough for the patient to be approachable or the surrogate to have recovered from the initial shock. Similar to prior consent, deferred consent may be opt-in or opt-out and the permissions given can vary in specificity (Table 2).

#### Deceased patients

Obtaining consent is especially problematic for the large majority of patients who do not survive the SCA episode. Should consent be sought from a representative in those cases or is an exception from consent justifiable? There is a lack of literature on this issue. Also, the European data protection law framework is not helpful since the GDPR does not apply to the personal data of deceased persons, although “Member States may provide for rules regarding the processing of personal data of deceased persons” (Recital 27). The majority of international guidelines do not address the issue of deceased patients or give conflicting advice [61].

The case against approaching relatives is the emotional stress this might cause, which conflicts with the healthcare professionals’ prima facie duty to prevent suffering and which could impact the validity of the consent [52]. When it concerns genetic information, however, family members might be said to have a stake as well. An Icelandic woman who objected to the use of her deceased father’s genetic data in the national Health Sector Database was backed by the Supreme Court based on the grounds that her own privacy was involved due to the direct biological link with her father’s DNA [62]. Moreover, disclosure of findings that have implications for relatives’ health becomes problematic when research was performed without their knowledge [63]. Whether relatives have similar rights as the original data subject, and how this should be dealt with in genetic research, is still an open question [62].

### Responsible data governance

When informed consent has been obtained, and even more so when research is conducted without consent, confidentiality of data should still be protected. Data governance policies should address privacy as well as broader societal issues (e.g. fairness) not covered by consent. We distinguish three main components of data governance: security and oversight, sharing and access and public engagement.

#### Data security and oversight

If personal data are fully anonymised, the GDPR does not apply. However, anonymising the data is not an ideal solution [64]. Firstly, it does not allow for control over usage of the information, i.e. data can be used for purposes that conflict with the data donor’s views. Secondly, the weaknesses of anonymization techniques and the uniqueness of genetic data prevent data from becoming truly de-identified. Instead, research data are often pseudonymised (‘pseudo-anonymised’), which means that additional information, stored elsewhere, is needed to link the personal data to a specific subject.

Still, other safeguards are needed to protect against unauthorised access and ensure integrity of research data (Article 89 of the GDPR). These may include secure storage within a robust information technology system, encryption of data and of identifiers used for linkage, access logging, the use of secure environments (‘data enclaves’), data access through a trusted third party or ‘honest broker’ and potentially new techniques such as block-chain [54, 65, 66]. Also, the GDPR requires the creation of a Data Protection Impact Assessment to evaluate privacy risks and adopt appropriate safety measures (Table 3). When deferred consent is used, there should be a pre-defined procedure for destruction of data from SCA victims who do not consent at the delayed time point or choose to withdraw from the study [67]. Another ‘safeguard’ is the oversight by the Research Ethics Committee or Data Protection Officer. In the absence of consent, the approval of an ethics committee is vital (Declaration of Helsinki, Article 32), especially when collecting human material (e.g. OECD Guidelines on Human Biobanks and Genetic Research Databases) [68] or performing data linkages [46, 56]. Moreover, as described by Morris et al. [21], obtaining approval from an ethics committee or privacy officer enhances the researchers’ credibility and thereby contributes to successful data collection from the various data sources.

**Table 3:** Selected key changes under the EU General Data Protection Regulation (GDPR)

Scope and definitions: • *Subject-matter*: The GDPR applies to the processing of personal data from natural persons (Article 1), thus excluding anonymous data (Recital 26) and data from deceased persons (Recital 27), as the ‘95 Directive did. • *Special categories*: Processing of data concerning health (Article 4(15)) and in the updated framework also genetic and biometric data (Article 4(13,14)) is in principle prohibited, unless one of the exceptions in Article 9(2) applies, e.g. when explicit consent has been provided (a) or when processing of sensitive data is necessary for scientific purposes (j) provided that safeguards are in place (Article 89(1)). • *Extended territorial scope*: The GDPR applies to all processing of personal data of EU citizens, whether it takes place in the EU or not (Article 3). Transfer of data to countries outside the EU may take place when the European Commission has evaluated the level of protection in the receiving country as adequate (Article 45), when appropriate safeguards have been provided (Article 46), or in case of specific derogations (Article 49). Principles and conditions for data processing: • *Principles*: The principles (Article 5) of data processing remain largely the same as those in the Directive: (a) lawfulness, fairness and transparency; (b) purpose limitation (note that secondary use of data for scientific purposes is presumed compatible with the original purpose (Recital 50)); (c) data minimisation; (d) accuracy; (e) storage limitation; (f) integrity and confidentiality. The principle of accountability (Article 5(2)), which holds that the data controller should be able to demonstrate compliance with the principles, has been added. • *Conditions for consent*: Data subjects’ consent (Article 4(11)) has become bound by stronger conditions in the GDPR (Article 7). When consent is used as the legal basis for processing, it should be “clearly distinguishable” from other matters and presented in an accessible form using clear and plain language. The controller should be able to demonstrate that consent was given, and the data subject is free to withdraw at any time. In the context of data processing for scientific research, the law leaves room for broad consent (Recital 33). Rights and responsibilities: • *Data subjects’ rights*: The GDPR introduces the right to data portability (Article 20), which allows transmission of one’s data to another controller. Moreover, the GDPR enhances existing rights, namely the right to: receive transparent information (Articles 12–14); access data (Article 15); rectification (Article 16); erasure (‘right to be forgotten’) (Article 17); restriction of processing (Article 18); object (Article 21); not to be subject to automated decision taking, including profiling (Article 22). However, in the context of scientific research Member States may provide derogations from these rights if they would impair research (Article 89). • *Privacy by design*: The idea of “data protection by design” is introduced to ensure risks are accounted for early through technical and organisational protective measures (Article 25). Processing of data for research purposes requires “appropriate safeguards” (Article 89(1)), although it is not specified what these should be. • *Data breaches*: Data controllers are required to keep a detailed record of all processing activities (Article 30) and in particular of any data breaches, which should be reported to the competent authorities within 72 h and—in case of high risk—to the data subject without undue delay (Articles 33 and 34). • *Data protection impact assessment*: For high risk processing of data (which includes processing of special categories of data, e.g. health data), the GDPR mandates performing a data protection impact assessment (DPIA) in order to ascertain the risks relating to data subjects’ rights (Article 35). • *Data protection officer*: Research institutions are now required to install a data protection officer (DPO) who monitors compliance with the GDPR, provides advice on data processing, including the DPIA, and acts as the contact point for the supervisory authority (Articles 37–39). • *Penalties*: Organisations that do not comply with the GDPR can be fined up to 4% of annual global turnover or 20 million EUR, whichever is greatest (Article 83(5)).	

#### Data sharing and access

The sharing of research data is increasingly framed as a scientific and ethical imperative [69]. Data sharing is especially important in fields where large numbers are needed, such as SCA research. However, barriers to data sharing include the lack of incentives for researchers who originally collected the data or the regulatory fragmentation across Europe, which the GDPR aims to harmonise [65, 70]. Researchers sharing data should check whether the receiving party consists of bona fide researchers, there is a justified research need, ethics approval has been obtained and sharing is in line with conditions pre-specified in data transfer or access agreements [65]. They also need to provide patients with meaningful access rights to their personal data and respect other patients’ rights that potentially apply, such as the right to be forgotten (Table 3).

Despite high levels of public support for data sharing among public institutions, people generally do not want their data to be shared for commercial gain [71]. Worries of commodification may become voiced even after the subject’s death: think of Henrietta Lacks’s family who want to be compensated for the unauthorised use of her cancer cells (the HeLa cell line) in 1951, which led to numerous medical advances [72]. Similarly, the *care.data* initiative in the UK, a linked database of patient information, was abandoned due to ethical concerns related to sharing data with commercial actors outside the National Health Service without consent. This led to a “collapse of context”: patients felt that their privacy was violated when the data were taken out of the health context to benefit the country’s economy [73]. Thus, fostering trust in research does not involve the right of participants to control all information flows, but requires transparency and what Nissenbaum [74] has termed *contextual integrity*.

#### Public engagement

In studies without opt-in consent, researchers often emphasise communitarian values such as solidarity or altruism as a reason not to ask consent [75]. However, this needs to be supported by data from the SCA context. In the US, intervention studies in the emergency setting are required to engage in prior community consultation to assess the acceptability of the research. Similarly, there is a general need for empirical studies on patients’ views regarding ethics in observational emergency research and for involvement of patients and other stakeholders in the creation of SCA database research consortia. Indeed, data governance is increasingly conceptualised as “data democracy” [64] and this deliberative approach of involving stakeholders may complement the Research Ethics Committee review and help to legitimise acute illness data research.

## Conclusions

Research with observational data from SCA victims is vital to improving prevention and treatment of SCA. Since the use of personal data could harm patients and communities—although usually to a lesser extent than experimental studies—legitimate consent procedures and data governance policies are needed. However, in our systematic literature search we encountered a lack of empirical work on the ethical aspects specific to non-interventional database research in SCA and other emergency settings. We therefore present the retrieved articles, supplemented by general literature on the ethics of biomedical big data, as a thematic narrative. In this review article we identified ethical issues that SCA researchers should be mindful of, in terms of both potential harms and approaches to address these harms, and we highlighted current knowledge gaps. Especially the dearth of guidance from the literature and law regarding data collection from deceased SCA patients warrants attention. There is a need for a context-sensitive, transnational and empirically informed ethical framework regarding collection, use and sharing of data from those who are struck by SCA or other acute and critical conditions.

## Additional file

Additional file 1: Search strategy. Table containing the PubMed search string as an example of the literature search strategy. (DOCX 15 kb)

## Abbreviations

ESCAPE-NET: European Sudden Cardiac Arrest network towards Prevention, Education and New Effective Treatment
EU: European Union
GDPR: General data protection regulation
SCA: Sudden cardiac arrest
