# Supplementary material for: Ethical aspects of sudden cardiac arrest research using observational data: a narrative review
Source: Crit Care. 2018 Sep 13;22:212. doi: 10.1186/s13054-018-2153-3 (PMC6136218; doi:10.1186/s13054-018-2153-3)
Supplement: Supplementary file 1 — Search strategy. Table containing the PubMed search string as an example of the literature search strategy. (DOCX 15 kb) [file 13054_2018_2153_MOESM1_ESM.docx]

**Additional file 1**

Table S1 Search strategy (PubMed)

| **Set#** | **Search string** | **Results** |
| --- | --- | --- |
| 1 | ethics[mh] OR "ethics, research"[mh] OR "legislation as topic"[mh] OR “informed consent"[mh] OR "patient rights"[mh] OR "confidentiality"[mh] OR "privacy"[mh] OR “social discrimination”[mh] OR ethic*[tiab] OR moral*[tiab] OR legal*[tiab] OR discrimination*[tiab] | 569 166 |
| 2 | "observational studies as topic"[mh] OR "datasets as topic"[mh] OR "databases as topic"[mh] OR "registries"[mh] OR database*[tiab] OR dataset*[tiab] OR registry[tiab] OR “big data”[tiab] OR “data collection”[tiab] | 617 012 |
| 3 | "out-of-hospital cardiac arrest"[mh] OR "death, sudden, cardiac"[mh] OR "cardiopulmonary Resuscitation"[mh] OR "arrhythmias, cardiac/complications"[mh] OR cardiac arrest*[tiab] OR heart arrest*[tiab] OR sudden cardiac death*[tiab] OR sudden death*[tiab] OR cardiopulmonary arrest*[tiab] OR cardiopulmonary resuscitation*[tiab] OR asystole*[tiab] | 102 831 |
| 4 | "emergencies"[mh] OR "critical care"[mh] OR "evidence-based emergency medicine"[mh] OR “emergency medicine"[mh] | 99 954 |
| 5a | (#1 AND #2 AND #3) AND (English[Language]) | 184 |
| 5b | (#1 AND #2 AND #4) AND (English[Language]) | 207 |
